# Substrate Stabilized Charge Transfer Scheme In Coverage Controlled 2D Metal Organic Frameworks

**DOI:** 10.1002/smll.202500507

**Published:** 2025-02-17

**Authors:** Simone Mearini, Dominik Brandstetter, Yan Yan Grisan Qiu, Daniel Baranowski, Iulia Cojocariu, Matteo Jugovac, Pierluigi Gargiani, Manuel Valvidares, Luca Schio, Luca Floreano, Andreas Windischbacher, Peter Puschnig, Vitaliy Feyer, Claus Michael Schneider

**Affiliations:** ^1^ Peter Grünberg Institute (PGI‐6) Jülich Research Centre 52428 Jülich Germany; ^2^ Institute of Physics University of Graz Graz 8010 Austria; ^3^ Present address: Physical and Computational Sciences Directorate and Institute for Integrated Catalysis Pacific Northwest National Laboratory Richland WA 99354 USA; ^4^ Physics Department University of Trieste Trieste 34127 Italy; ^5^ Elettra – Sincrotrone Trieste S.C.p.A. S.S. 14 km 163.5 Trieste 34149 Italy; ^6^ ALBA Synchrotron Light Source Barcelona 08290 Spain; ^7^ CNR – Istituto Officina dei Materiali (IOM) TASC Laboratory Basovizza Trieste 34149 Italy; ^8^ Faculty of Physics and Center for Nanointegration Duisburg‐Essen (CENIDE) University of Duisburg‐Essen 47048 Duisburg Germany; ^9^ Department of Physics and Astronomy UC Davis Davis CA 95616 USA

**Keywords:** 2D materials, charge transfer, molecular ligands, single‐layer metal‐organic frameworks, transition metals

## Abstract

Recently, 2D metal‐organic frameworks (2D MOFs), characterized by complex charge transfer mechanisms, have emerged as a promising class of networks in the development of advanced materials with tailored electronic and magnetic properties. Following the successful synthesis of a 2D MOF formed by nickel (Ni) linkers and 7,7,8,8‐tetracyanoquinodimethane (TCNQ) ligands, this work investigates how the Ni‐to‐ligand ratio influences the electronic charge redistribution in an Ag(100)‐supported 2D MOF. The interplay between linker‐ligand and substrate‐MOF charge transfer processes leads to a stable equilibrium, resulting in a robust electronic structure that remains independent of stoichiometric ratios. This stability is primarily based on the electron transfer from the metal substrate, which compensates for charge imbalances introduced by the metal‐organic coordination across different MOF configurations. Despite minor changes observed in the magnetic response of the Ni centers, these findings emphasize the robustness of the electronic structure, which remains largely unaffected by structural variations, highlighting the potential of these 2D MOFs for advanced applications in electronics and spintronics.

## Graphical abstract

The synthetic process of 2D frameworks composed of nickel and 7,7,8,8‐tetracyanoquinodimethane on Ag(100) involves the formation of multiple phases. Two distinct architectures with atom: ligand ratios of 1:2 and 1:1 are studied with a multi‐technique approach and stable electronic properties are observed, except for slight differences in magnetic responses.

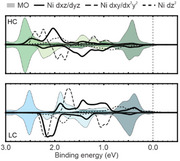

## 1 Introduction

The search for materials with tailored electronic and magnetic properties has driven extensive research in 2D systems. Specifically, 2D MOFs emerge as a versatile material class that offers a remarkable combination of molecular precision and solid‐state properties. In general, MOFs are composed of metal ions coordinated by organic ligands to form crystalline structures. Due to the wide variety of chemical compounds, systems with finely tuned properties can be synthesized. Traditionally, these frameworks find application in gas storage and catalysis.^[^ ^1^, ^2^, ^3^, ^4^, ^5^, ^6^, ^7^, ^8^, ^9^, ^10^ ^]^ Furthermore, 2D MOFs are now being explored for their promising capabilities in electronics.^[^ ^11^, ^12^, ^13^, ^14^ ^]^ Moreover, they provide the possibility to introduce a bandgap through strategic chemical design, a feature absent in pristine graphene.^[^ ^4^, ^11^, ^15^, ^16^ ^]^ Finally, the extensive π‐conjugation and the potential for high carrier mobility make these materials promising for next‐generation electronic devices.^[^ ^1^, ^17^, ^18^, ^19^, ^20^ ^]^

The electronic structure of 2D MOFs is fundamentally governed by the hybridization between the orbitals of the metal linkers and the organic ligands. The emergence of such hybrid states goes hand in hand with a strong delocalization of electrons, significantly determining the electronic properties of the framework. This has been demonstrated for 2D MOFs composed of Ni atoms and 1,2,4,5‐tetracyanobenzene (TCNB) molecules deposited on metal substrates, where the origin of new energy dispersive states has been traced back to the mixing of Ni 3d states and the π‐symmetric frontier molecular orbitals (MOs) of TCNB.^[^ ^21^ ^]^ Furthermore, it has been demonstrated that the energy level of these hybrid states and, consequently, the electronic properties of the MOFs can be tuned by incorporating different transition metal (TMs) centers into their structure.^[^ ^22^ ^]^

Although significant progress has been made in the bottom‐up manufacturing of 2D MOFs over the past decade, ensuring the structural stability of the network remains a critical step for industrial applications. In such scenarios, the 2D MOFs are typically supported on solid electrodes, which not only provide mechanical stability but can also actively influence their electronic properties. In the bottom‐up synthesis process, the long‐range order of the MOF is primarily determined by the sequential deposition of ligand molecules and linker atoms on metal substrates. During this process, the assembling MOF may undergo several phase transitions depending on the stoichiometric ratio of its components and the substrate nature, which acts as a template for the MOF formation. Metal‐organic coordination, which arises from the hybridization between the orbitals of the TM nodes and the organic ligands, governs the intrinsic properties of the MOF. Nevertheless, the fine interplay in the electron transfer processes, mediated by the TM centers or the substrate, can alter the electronic structure and enhance the robustness of the electronic and magnetic properties of the frameworks. Therefore, understanding this interplay is the key to unlock the full potential of MOFs, but it still remains poorly understood.

To address this gap, this work investigates how different stoichiometric ratios of ligand molecules and central TM atoms affect the electronic and magnetic properties of the MOF layers. Specifically, the study focuses on 2D MOFs composed of Ni atoms and TCNQ molecules deposited on an Ag(100) surface. The choice of TCNQ as an organic ligand is motivated by two key factors. First, the variation of Ni concentration gives control over the formation of two distinct phases with well‐defined stoichiometric Ni:TCNQ ratios. Secondly, TCNQ possesses a high electron affinity and thus tends to attract electrons from close‐by partners, increasing its aromaticity.^[^ ^23^, ^24^ ^]^ These characteristics make this molecule particularly well suited to study the charge transfer phenomena occurring in 2D MOFs with variable Ni concentration, where Ni atoms actively participate in redox reactions with TCNQ.

To explore these phenomena, we employ a combination of experimental and theoretical surface science techniques for a comprehensive study of these MOF phases. Low energy electron diffraction (LEED) provides insight into structural properties, while near‐edge X‐ray absorption fine structure (NEXAFS) and photoemission valence band (VB) spectroscopy examine the electronic characteristics of the MOF layers. Furthermore, X‐ray magnetic circular dichroism (XMCD) experiments probe the presence of individual and collective magnetic behavior of the central metal nodes. These findings are then corroborated and explained by density functional theory (DFT) calculations.

Our results conclusively demonstrate a robust electronic structure across the phases involving a single electron donation from each Ni atom to the former lowest energy unoccupied molecular orbital (LUMO) of TCNQ. When varying the Ni:TCNQ stoichiometry, the concomitant changes in the total number of electrons involved in this redox reaction are, however, compensated by the electron transfer from the Ag substrate, which actively stabilizes the framework. Consequently, strongly dispersive hybrid states, that are formed between the frontier MOs and the Ni 3d states and emerge in both phases, remain largely unaffected by the geometry and stoichiometry. Furthermore, magnetization measurements hint at the presence of collective magnetic behavior of the metal centers mediated by superexchange interactions, which become slightly stronger at higher Ni concentrations within the MOF structure.

## 2 Results and Discussion

**Figure 1:**
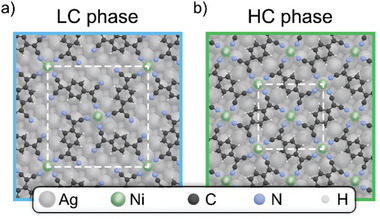
Real space representation of the energetically most stable unit cells for a) LC and b) HC phase in the top‐hollow adsorption geometry, respectively.

The sublimation of TCNQ molecules onto an Ag(100) substrate results in an ordered TCNQ self‐assembled monolayer (TCNQ‐SAM).^[^ ^25^ ^]^ Subsequent adsorption of Ni leads to the formation of a MOF with a stoichiometric Ni_2_‐(TCNQ)_4_ composition, where each Ni atoms is coordinated to four cyano (CN) groups. In this structure, half of the CN groups of each TCNQ molecule coordinates to the neighboring Ni atoms in a trans‐pattern. The other half of the CN groups remains unbounded. This structure is referred to as the “low coverage” (LC) phase.^[^ ^25^, ^26^, ^27^ ^]^ With further Ni deposition, a second phase emerges with a balanced Ni_2_‐(TCNQ)_2_ composition, referred to as the “high coverage” (HC) phase.^[^ ^25^ ^]^ In this phase, all CN groups bond to surrounding Ni atoms, which preserve the same coordination environment, and achieve a complete saturation of the ligand molecules. In this study, the structural models of both phases were determined through a complementary process, starting from published STM data^[^ ^25^ ^]^ and our LEED results, in combination with computational modeling. The resulting structures are depicted in **Figure** **1**. Additional Ni deposition beyond the HC phase leads to the formation of metal clusters on the substrate, consistent with observations in similar systems.^[^ ^28^ ^]^

Former STM studies^[^ ^25^ ^]^ indicate that both MOFs adopt a “top‐hollow” adsorption configuration, where the Ni atoms alternate between sitting directly on top of the Ag atoms and on the hollow sites in between. Our DFT calculations indicate that the HC phase rather exhibits a slight preference for a “bridge‐bridge” configuration over the abovementioned “top‐hollow” arrangement, with a marginal energy difference of −0.017 eV per TCNQ molecule. The opposite is true for the LC phase, where the “top‐hollow” configuration is drastically favored. In the “bridge‐bridge” configuration, all Ni atoms are positioned on a nearest‐neighbor bridge site between two Ag atoms of the first layer.

The detailed theoretical investigation of both configurations of the HC phases indicates that the difference in the adsorption geometry has a negligible impact on the electronic structure of the MOF. Consequently, the following discussion will focus exclusively on the “top‐hollow” configuration for both LC and HC phases, as it effectively captures the essential properties of the system.

The two structures correspond to a commensurate epitaxy of (5, 4; −4, 5) for the LC phase and (4, −1; −1, −4) for the HC phase, with a cell length of 18.4 Å and domain orientation ±38.7° respect to [011] for the LC phase and a cell length of 11.8 Å and domain orientation ±14.0° respect to [011] for the HC phase.^[^ ^25^ ^]^ These unit cells are validated through our LEED measurements, shown in Figure S1 (Supporting Information), which can be accurately replicated by LEED simulations using the above‐mentioned matrices.^[^ ^25^ ^]^

**Figure 2:**
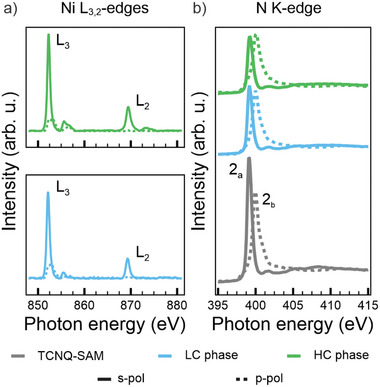
a) Ni L_3,2_‐edge NEXAFS spectra measured with s‐ (solid line) and p‐ (dashed line) polarized light for the LC phase (bottom) and HC phase (top); “L_3_” and “L_2_” indicate the main absorption peaks; b) NEXAFS spectra acquired across the N K‐edge with s‐ (solid line) and p‐ (dashed line) polarized light; from bottom to top: pristine TCNQ‐SAM (gray), LC (green) and HC (cyan) phase; “2_a_” and “2_b_” indicate the main absorption peaks.

Having established the structural characteristics of the MOFs, we conduct NEXAFS measurements across the Ni L_3,2_‐edges to gain deeper insights into the electronic properties of the Ni linkers. **Figure** **2**a shows the resulting spectra for s‐ (solid lines) and p‐ (dashed lines) polarized light, with the LC phase (cyan) on the bottom and the HC phase (green) at the top.

The NEXAFS spectra, in general, and the linear dichroic behavior, in particular, show a strong resemblance to those previously observed for Ni tetraphenylporphyrin (NiTPP) on metal substrates.^[^ ^29^, ^30^, ^31^ ^]^ Spectra collected with s‐polarized light show two distinct peaks at 852.3 and 869.5 eV corresponding to the L_3_ and L_2_ edge transitions, respectively. Each resonance is accompanied by a multicomponent satellite feature at 2.5–3.0 eV higher energy. From comparison with the literature,^[^ ^30^ ^]^ these spectra correspond to the L_3_ and L_2_ resonances of Ni(I), indicating a d^9^ electron configuration for the metal ion.

The leading sharp resonances display a pronounced linear dichroism, corresponding to differences in spectral intensity between measurements with s‐ and p‐polarized light, which provides insights into the occupancy of the 3d orbitals in the Ni(I) centers. As shown in the structural models reported in Figure 1, each Ni atom experiences a ligand field of D_4__h_ symmetry. According to crystal field theory, the ligand field induces an energy splitting of the five Ni d‐states with the in‐plane $d_{x^{2}-y^{2}}$ orbital ending up highest in energy due to the maximized spatial overlap with the charge density from the ligand molecules. The distribution of the nine d‐electrons will, consequently, leave an unpaired electron in the σ‐symmetric $d_{x^{2}-y^{2}}$ orbital. This explains the aforementioned linear dichroic behavior in the spectra, as s‐polarized light is required for observing the excitation into a vacant σ‐symmetric state.^[^ ^32^, ^33^, ^34^, ^35^ ^]^

Based on our analysis, the Ni linker is oxidized from its atomic configuration d^10^ Ni(0) to d^9^ Ni(I), suggesting a charge redistribution upon coordination into the organic framework. The in‐plane σ‐symmetry of the Ni $d_{x^{2}-y^{2}}$ orbital prevents a direct charge transfer from the Ni ion to the Ag atom underneath (top site), based on symmetry arguments.^[^ ^36^ ^]^ Although charge transfer from the Ni ions positioned on the hollow sites toward the substrate is symmetry‐allowed, it can be excluded due to the absence of Ni(0) ions alongside the detected Ni(I) oxidation state.^[^ ^36^ ^]^ As a consequence, the Ni electron can be solely donated to the molecular ligands.

To further investigate this phenomenon, NEXAFS spectra were recorded with s‐ (solid line) and p‐ (dashed line) polarized light across the N K‐edge for the LC phase (cyan lines), the HC phase (green lines) and the TCNQ‐SAM (gray lines), as shown in Figure 2b. We remark the absence in any spectrum of the characteristic lowest energy resonance, typically observed at ≈397.5 eV in the reference NEXAFS spectra acquired for the TCNQ powder, indicating the complete filling of the LUMO.^[^ ^23^, ^37^, ^38^ ^]^ In particular, the spectral behavior in Figure 2b suggests that a significant charge transfer from the Ag(100) substrate to the TCNQ molecules already occurs in the pristine TCNQ layer, prior to Ni deposition. The N K‐edge spectra exhibit two prominent resonances at 399.2 and 400.0 eV for s‐ and p‐polarization, respectively. These peaks, labeled 2_a_ and 2_b_, correspond to two orthogonal π^*^‐symmetry orbitals of the CN groups, which are degenerate in the isolated radical. Both orbitals are orthogonal to the CN bond axis, with one oriented parallel (2_a_) and the other vertical (2_a_) with respect to the molecular plane.^[^ ^32^, ^33^, ^34^ ^]^ The perfect linear dichroism observed for the two π^*^‐symmetry resonances in the pristine TCNQ layer indicates a preferential orientation of the CN groups parallel to the Ag(100) substrate. Upon adsorption of the Ni atoms into the TCNQ‐SAM, a slight decrease in relative intensities of these resonances is observed. Specifically, after Ni deposition (for both LC and HC phases), the resonance at 400.0 eV retains its perfect dichroism, vanishing in s‐polarization, which confirms that its π^*^ orbital remains perpendicular to the surface. In contrast, the resonance at 399.2 eV displays increased residual intensity in p‐polarization with respect to the pristine TCNQ‐SAM, indicative of a small rotation of the CN groups. Despite these changes, the energy positions and widths of both resonances remain unaltered. This invariance implies that direct charge transfer from the Ni ion to the corresponding MOs of TCNQ is unlikely. The orthogonality of these π^*^‐symmetry orbitals relative to the ligand‐ion bond renders such transitions symmetry‐forbidden. Consequently, we conclude that the electrons released by the Ni ions are instead accepted by the LUMO of TCNQ.

Summarizing the interpretation of the NEXAFS data, we propose the following charge rearrangements in both MOF systems. Upon assembling the TCNQ‐SAM, the LUMO of the molecule gets filled via charge transfer from the Ag substrate. The overall occupation of this MO remains unchanged after the addition of Ni. Importantly, as Ni also acts as an electron donor to the molecule, we may conclude that the amount of electron transfer from the substrate to the LUMO of TCNQ progressively decreases as the electron transfer from Ni into the same MO increases, the latter following the increasing Ni coverage from the LC phase to the HC one.

**Figure 3:**
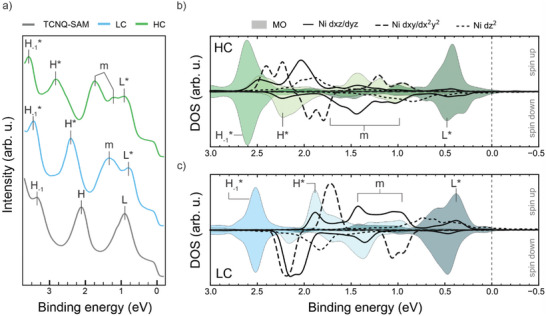
a) Experimental VB spectra (photon energy 30 eV, p‐polarized light) acquired for the TCNQ‐SAM (gray line), LC (cyan line), and HC (green line) phase, respectively; “L”, “H”, and “H‐1” indicate the LUMO, HOMO and HOMO‐1 peaks, respectively; the “*” on top indicates the respective hybrid states with additional Ni contributions; “m” refers to the states with mixed contributions from multiple MOs; b) and c) calculated DOS projected on the Ni 3d states (black lines) and the TCNQ MOs (shaded areas) for the HC and LC phases, respectively.

To further corroborate these findings, in the next step, we investigate the electronic structures of the MOF phases by VB photoemission spectroscopy. This well‐established technique probes the density of states (DOS) near the Fermi level of single‐layer 2D materials and, has recently been employed to study metal‐organic hybridization within MOFs.^[^ ^8^, ^21^, ^22^, ^35^, ^39^, ^40^, ^41^, ^42^ ^]^ **Figure** **3**a shows the VB spectra of the pristine TCNQ‐SAM, as well as those of the LC and HC phases. The spectrum of the TCNQ‐SAM (gray line) exhibits features consistent with those reported in previous studies.^[^ ^43^ ^]^ This includes a peak at a low binding energy (*BE*) of ≈0.90 eV, attributed to the filled LUMO (L), and higher *BE* features at 2.10 and 3.30 eV, corresponding to the HOMO (H) and HOMO‐1 (H_‐1_) orbitals, respectively. Notably, the presence of the LUMO feature in the TCNQ‐SAM VB spectrum underlines the charge donation from the substrate to the adsorbed molecules, in agreement with the interpretation of the absent resonance in N K‐edge NEXAFS spectrum discussed above.

Upon depositing Ni onto the pristine TCNQ‐SAM, the electronic structure changes for both the LC and HC phases. Specifically, new electronic states, labeled with an “m”, appear in the [2.00, 0.50] eV *BE* region. As will be explained in more detail below, these states arise from the hybridization between the Ni 3d states and the TCNQ MOs, mainly involving the HOMO and LUMO, as previously discussed for similar systems.^[^ ^21^, ^22^, ^27^, ^44^, ^45^ ^]^

Furthermore, a global shift of the molecular peaks to higher *BE* is evident, with a shift of *Δ BE* = 0.09 eV from the TCNQ‐SAM to the LC phase, and *Δ BE* = 0.14 eV from the LC phase to the HC phase. This shift can be rationalized by the decreasing amount of charge transfer from Ag into the former LUMO of the MOF. Consequently, this leads to a lowering of the Fermi edge with respect to the vacuum level, which is reflected in an increase in *BE*. Work function (*Φ*) measurements, reported later in **Table** **1**, further support this interpretation.

**Table 1:** Experimental and simulated Φ_*rel*_ values, evaluated as the difference between the framework and the Ag(100) work functions, for the studied systems.

Systems	Φ_*rel*,*exp*_ [eV]	Φ_*rel*,*sim*_ [eV]
Ag(100)	0.00	0.00
TCNQ‐SAM	+0.53	+0.66
LC phase	+0.37	+0.52
HC phase	+0.11	+0.36

While for the TCNQ‐SAM we observe a 0.53 eV higher work function compared to atomically clean Ag(100), this relative value then decreases to +0.37 and +0.11 eV for the LC phase and HC phases, respectively.

Although the two MOF phases show very similar behavior in terms of electronic configuration, this *BE* shift is particularly pronounced for the H^*^ peak, which additionally indicates an energy broadening. As such behavior cannot be explained by a mere charge transfer from the substrate, we tentatively attribute it to changes in the character of the hybrid states involving the HOMO.

We have conducted comprehensive DFT calculations to investigate such hybridization phenomena between the 3d states of Ni and the TCNQ MOs within the MOF systems, and how they change when going from the LC to the HC phase. The structural models of both MOF phases are fully relaxed, incorporating five layers of the supporting Ag(100) substrate using a repeated slab approach. The resulting optimized geometries have already been depicted in Figure 1. Note that all calculations are performed using a van der Waals corrected (DFT‐D3) generalized gradient approximation exchange‐correlation functional (PBE‐GGA) Importantly, a DFT+U self‐interaction error correction (*U* = 3 eV) is applied to compensate the self‐interaction error in the Ni d‐orbitals. Further methodological details are provided in the Supporting Information.

Figure 3b,c show the projected density of states (PDOS) onto the 3d states of Ni (black lines) in the HC (b) and LC (c) phase, where we follow the convention that positive (negative) binding energies refer to occupied (unoccupied) states. Also note that for clarity, the projections of the degenerate *d_xz_* and *d_yz_* states, as well as those of the degenerate in‐plane *d_xy_* and $d_{x^{2}-y^{2}}$ states, are combined and, outside the displayed *BE* range, there are some non‐vanishing contributions of $d_{xy}/d_{x^{2}-y^{2}}$. In particular, a significant contribution from the $d_{xy}/d_{x^{2}-y^{2}}$ projection is observed above the Fermi level (see Figure S2, Supporting Information). This indicates a single integer charge donation from the $d_{xy}/d_{x^{2}-y^{2}}$ leaving the Ni in a d^9^ state for both LC and HC phases, which aligns well with the Ni L_3,2_‐edges NEXAFS data discussed above (see Figure 2a).

To gain deeper insight into the electronic structure of the ligand molecules, the total density of states is projected onto individual molecular orbital (MOPDOS), as shown in Figure 3. First, Figure 3 confirms the complete occupation of the LUMO for all the TCNQ molecules (indicated by the shaded area) in both the LC and HC phases, in agreement with the experimental NEXAFS data for the N K‐edge (Figure 2b). Second, the analysis also reveals significant hybridization between the symmetry‐compatible out‐of‐plane *d_xz_*/*d_yz_* and the π‐symmetric molecular frontier orbital (HOMO and LUMO) in the *BE* range of [0.50, 2.50] eV. This type of hybridization has already been reported in previous works.^[^ ^21^, ^22^ ^]^ In contrast, the HOMO‐1 is localized almost entirely on the benzene ring and, therefore, does not significantly interact with the Ni atoms, leading to sharp, unhybridized projection peaks. Thirdly, using this sharp feature as a reference, the MOPDOS supports the observed trend of an overall shift toward higher *BE*s going from the LC phase to the HC phase (*Δ BE* = 0.18 eV) and a corresponding decrease of the work function of 0.20 eV. Further support for this interpretation comes from the overall good agreement between the experimental and calculated work functions reported in Table 1 (see also Figure S3, Supporting Information for a more detailed analysis of the work function data).^[^ ^46^, ^47^ ^]^

In order to trace back the origin of the observed work function changes and to quantify the amount of transferred electrons from the substrate, we analyze the charge density averaged over planes parallel to the substrate surface as a function of the coordinate z perpendicular to the surface.

**Figure 4:**
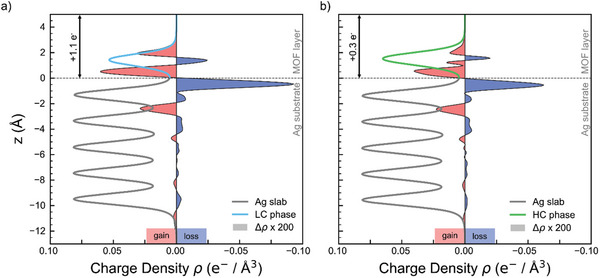
Plane‐averaged total charge density *ρ* (lines), normalized by the area spanned by the surface‐parallel vectors of the unit cell, and charge density difference *Δρ* (shaded area; scaled by a factor of 200) as a function of the height z parallel to the interface normal for the a) LC and b) HC phase, respectively. The red and blue areas indicate regions with an overall gain or loss in electrons compared to the bare substrate / freestanding MOF layer, respectively.

**Figure** **4** shows the total charge density *ρ* (lines), normalized by the area spanned by the surface‐parallel vectors of the unit cell, as well as the charge density difference *Δρ* (shaded areas, scaled by a factor of 200) between the entire substrate‐MOF interface compared to the bare substrate and freestanding MOF layer for the LC (a) and HC (b) phases, respectively. The five gray peaks below the substrate‐MOF boundary at *z* = 0 are indicative of the five Ag layers in our model slab, whereas the peak of the colored curve for *z* = 0 is due to the LC (blue) and HC (green) MOF layers. When now focusing on the charge density difference (red‐shaded areas indicating electron accumulation, blue‐shaded areas electron depletion), a charge donation from the topmost Ag layer into the MOF with a subsequent redistribution of charge inside the slab to compensate becomes immediately apparent. Integration of this charge density difference above the interface boundary results in a total charge donation of +1.1 e^−^ and +0.3 e^−^ from the substrate to the MOF for the LC and HC phases, respectively. While a definite assignment of charge density to individual parts of a structure is notoriously tricky owing to the, to some degree, arbitrary definition of corresponding spatial regions, the decrease in charge donation by roughly a factor of 3 between the LC and HC phases is in qualitative accordance with our stoichiometric analysis, reported earlier. In the HC phase, the higher Ni density leads to an increased amount of charge transferred from Ni to TCNQ, minimizing the need for substrate donation. Accordingly, in the LC phase, the presence of fewer Ni centers results in reduced Ni‐to‐TCNQ charge transfer, necessitating greater substrate contribution to fully occupy the TCNQ LUMO and stabilize its charge state. This balance between Ni‐to‐TCNQ and substrate‐to‐MOF charge transfer explains the higher charge donation observed for the LC phase. The Bader charge partition scheme^[^ ^48^ ^]^ further corroborates this threefold decrease in charge donation between the LC and HC phases.

Overall, there is an excellent agreement between experimental and theoretical data across all major points, supporting the robustness of the electronic structure regardless of the geometry or stoichiometry of the MOF phase.

After having understood the electronic structure of the MOFs, we focus on the magnetic properties of the Ni centers in both the LC and HC phases. Although the electronic structure of the Ni centers in both MOF phases is similar, it is important to verify whether the different Ni:TCNQ ratio affects the magnetic properties. Orbital overlap between neighboring magnetic ions leads to direct exchange interactions; a super exchange mechanism, mediated by hybridization between d‐orbitals of TM atoms and orbitals of the ligand, can further contribute to magnetism.^[^ ^26^, ^45^ ^]^ In the MOF structures, these superexchange interactions are crucial for mediating magnetic coupling between the metal centers, leading to a collective magnetic behavior within the MOF systems.

**Figure 5:**
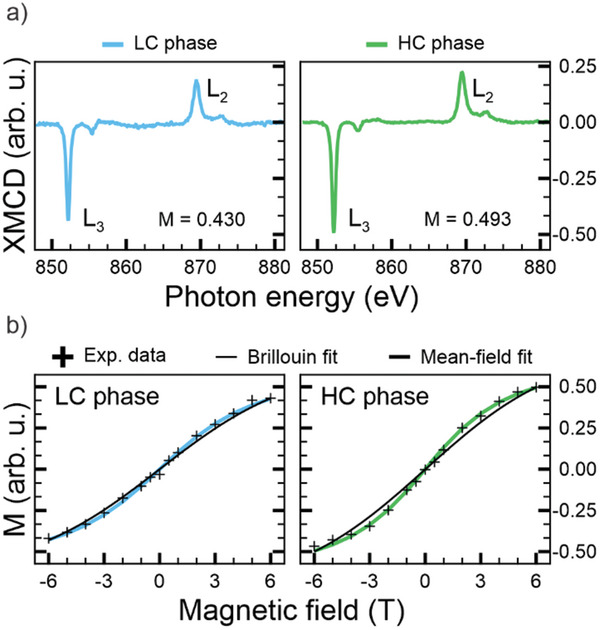
a) XMCD spectra, generated as the difference between the XAS spectra acquired at the Ni L_2,3_‐edges with opposite circularly polarized light (Figure S4, Supporting Information), of the LC (left) and HC (right) phase obtained at normal incidence geometry (*T* ≈ 5 K, *B* = 6 T); b) magnetization curves for the LC (left) and HC (right) phase under an external magnetic field in the range [−6, 6] T; the black crosses indicate the experimental data, corresponding to the L_3_‐edge maximum signal intensity of each XMCD spectra acquired at 5 K under different external magnetic fields; the black curve represents the Brillouin fit for *S* = 1/2; the cyan and green curves are the fits obtained with the mean‐field approach with *W* equal to 0.015 eV for the LC and HC Ni MOFs, respectively.

To this end, we conduct XMCD experiments for both frameworks. The X‐ray absorption spectra (XAS), acquired with left (*I* ^−^) and right (*I* ^+^) circularly polarized light across the Ni L_3_,_2_‐edges under an applied magnetic field *B* = 6 T and at a temperature of ≈ 5 K, are presented in Figure S4 (Supporting Information). The corresponding XMCD spectra, defined as (*I* ^− ^ −  *I* ^+^) and normalized by the value of (*I* ^−^ +  *I* ^+^) at the maximum of the L_3_ edge, are shown in **Figure** **5**a. In both metal‐organic structures, the coordinated Ni ions exhibit sizeable magnetic moments. Remarkably, as the MOF structures transform from the LC to the HC phase, the XAS and XMCD spectra display similar spectral features with peaks occurring at the same photon energies. This observation further corroborates the earlier discussion regarding the stabilization of Ni centers predominantly in a d^9^ electronic configuration for both MOF phases. The presence of an unpaired electron in the d‐shell suggests that the material exhibits paramagnetic behavior. Despite the identical electronic configuration of the Ni atoms in both phases, the XMCD spectra reveal a small, but noticeable, difference of ≈ 13% in magnetization (*M*) values under an applied magnetic field of 6 T, with the HC phase showing a slightly higher magnetization.

To better understand this difference, the magnetic properties of the systems are analyzed through magnetization curves, shown in Figure 5b. The *M* values are evaluated as the maximum signal intensity of the L_3_ edge for each XMCD spectra acquired under different external magnetic fields (*B*), in the range [−6, 6] T, while the temperature is kept at *T* = 5 K. To rationalize the experimental *M* versus *B* trend at a given temperature, the Brillouin function is applied, which provides the analytical expression for magnetization curves in a system with a specific spin quantum number (*S*).^[^ ^41^, ^42^ ^]^ For a Ni(I) ion with *S* = 1/2 and at *T* = 5 K, the fitting procedure generates the thinner black curves reported in Figure 5b for both MOFs. However, the discrepancy between the experimental results and the *S* = 1/2 model suggests that magnetic interactions between individual Ni centers in the MOFs, likely mediated by the TCNQ ligands via a superexchange mechanism, should be considered to fully describe the magnetic behavior of the systems.^[^ ^26^, ^45^ ^]^

A mean‐field‐like fitting approach is applied to the experimental data to account for this additional interaction and quantify the exchange coupling constant *J*. In this model, each Ni(I) ion is assumed to be in a square planar coordination environment with a localized spin *S* = 1/2 in its partially filled $d_{x^{2}-y^{2}}$ orbital. The fitting procedure, based on Weiss's theory (more details in the Supporting Information), couples molecular spin with the mean‐field itinerant spin density via an effective exchange coupling constant *J*.^[^ ^45^ ^]^ Assuming a bandwidth *W* = 0.015 eV and a g‐factor of 2, the resulting fits, reported in Figure 5b (cyan and green curves for the LC and HC phase, respectively), are in good agreement with the experimental trend and lead to *J* values of 0.9 and 1.1 meV for LC and HC phase, respectively.^[^ ^26^, ^45^ ^]^

These estimated *J* values align with those reported for similar systems.^[^ ^26^, ^45^ ^]^ The subtle yet measurable difference in the magnetization curves of the LC and HC phases can be attributed to variations in the strength of the super exchange mechanism within the MOF layer. While both phases exhibit similar electronic structures, their geometric differences impact the number of direct ligand‐metal center bonds. In the LC phase, half of the CN groups on TCNQ remain uncoordinated, whereas in the HC phase, complete ligand saturation enhances connectivity between metal centers. This stronger connectivity likely reinforces super exchange interactions, leading to a more collective magnetic response, as evidenced by the steeper slope of the magnetization curve in Figure 5b compared to Figure 5a.

While the magnetization curves provide strong evidence for ligand‐mediated super exchange, further magnetic investigation could offer deeper insights into the magnetic properties of Ni‐TCNQ MOFs.

## 3 Conclusion

In this work, a combined effort of DFT calculations and synchrotron‐based spectroscopic methods is applied to investigate the electronic and magnetic properties of 2D MOFs while varying the TM concentration. Specifically, two phases of Ni‐TCNQ MOFs deposited on Ag(100), with Ni : TCNQ stochiometric ratios of 1:2 and 1:1, respectively, are studied. In both phases, a stable d^9^ Ni(I) electron configuration is observed for all Ni ions and the complete occupation of the former LUMO of all TCNQ ligands is confirmed. This behavior is attributed to a redox reaction between the Ni centers and the organic ligands, involving a single integer charge transfer of the highest‐lying Ni 3d orbital to the TCNQ molecules, regardless of the stoichiometry of the phase. The remaining charge necessary to fully occupy the LUMO orbitals, a number which depends on the stoichiometry, originates from the metallic substrate, which contributes then to maintaining the constant electronic properties of the system at different ligand saturation levels.

The electronic charge rearrangements induced by variations in ligand saturation are accompanied by subtle changes in the magnetic properties of the Ni linkers. Magnetization curves reveal deviations from the expected Brillouin model, which are attributed to a superexchange mechanism mediated by the TCNQ ligands. This interaction contributes to the magnetic behavior, further emphasizing the role of ligand‐mediated coupling in modulating the magnetic response of the system.

Our findings underline the robustness of electronic and magnetic properties demonstrated by this exciting new class of materials. Combining the versatility provided by the choice of constituents with the stability of their properties throughout their formation makes 2D MOFs excellent candidates for the next generation of miniature electronic and spintronic devices.

## 4 Experimental Section

### Sample Preparation

The Ag(100) crystal was cleaned using standard methods involving ionized argon (Ar^+^) sputtering and heating to 800 K. TCNQ was evaporated onto the crystal at 400 K, while Ni was subsequently deposited onto the substrate while maintaining the sample at 540 K using an e‐beam evaporator. The formation of the LC and HC metal‐organic phases was confirmed using a LEED technique, which was also utilized to adjust the deposition rate for Ni within the multi‐technique approach presented in this study.

It is worth noting that the stabilization of the fully saturated and commensurate HC Ni MOF phase, which is characterized by the square lattice reported in Figure 1b, benefits from the heating of the sample to temperatures of ≈ 540 K during the Ni metal deposition. Annealing to lower temperature favors instead a mixed phase in which the abovementioned architecture coexists with an incommensurate phase with rectangular unit cell, referred to as β phase by Tseng et al.^[^ ^25^ ^]^ Additionally, heating the sample to temperatures higher than 630 K induces the MOF decomposition, as evidenced by the partial reduction of intensity of the LEED spots in Figure S5 (Supporting Information). At 670 K, the total absence of distinct features in the LEED experiments indicates the total decomposition of the MOF, confirming the stability of the HC phase up to 630 K.

Figure S5 (Supporting Information) also suggests that the stability of the LC Ni MOF is significantly lower. New spots begin to appear at 590 K, and two orderings (HC and LC) start to coexist. Further heating to 630 K results in the new phase becoming dominant, and by 640 K, no clear features are present in the LEED image, indicating complete disruption of the MOF.

### ARPES Measurements

The ARPES experiments were conducted at the NanoESCA beamline of the Elettra synchrotron in Trieste, Italy, using a photoelectron emission microscope (PEEM).^[^ ^49^ ^]^ Both the VB photoemissions spectra and the WF scans (acquired across the secondary electrons cut‐off) were obtained by setting the photon energy at 30 eV and using p‐polarized light. The measurements were performed at a pressure below 1 × 10^−10^ mbar with the sample cooled to 90 K using an open‐cycle cryostat. The total energy resolution was 100 meV and the momentum resolution of the PEEM was ± 0.05 Å^−1^. To ensure data quality and avoid radiation damage on the molecular‐based frameworks, the samples were rastered during measurements.

### NEXAFS Measurements

NEXAFS measurements were conducted at the ALOISA beamline of the Elettra synchrotron in Trieste, Italy.^[^ ^50^ ^]^ Spectra were collected using partial electron yield mode with a channeltron and a polarized grid. The linear polarization with respect to the scattering plane was switched from Transverse Electric (strictly s‐polarization) to Transverse Magnetic (almost p‐polarization) by rotation of the sample around the photon beam axis (manipulator coaxial to the photon beam), while keeping the surface at constant grazing angle of 6°. Normalization and energy calibration followed a protocol described in the reference.^[^ ^51^ ^]^ The experiment was conducted at a pressure below 1 × 10^−10^ mbar, with the sample maintained at 300 K.

### XMCD Measurements

The XMCD experiments were performed at the BOREAS beamline at the ALBA synchrotron.^[^ ^52^ ^]^ Magnetic field and temperature conditions for XMCD data collection were set at *B* = 6 T and T ≈ 5 K. For the magnetization experiments, the magnetic field was varied in the range [6, ‐6] T, while maintaining *T* ≈ 5 K. The measurements were conducted at the Ni L‐edge, with the magnetic field fixed in the direction of the incident light. The spectra were collected using total electron yield mode. To prevent beam damage, the sample was continuously moved to access non‐illuminated fresh sample spots.

The magnetization curves signal was defined as the difference between the intensities of left‐ and right‐circularly polarized light (*I* ^− ^ −  *I* ^+^) at the maximum of the Ni L_3_ edge, normalized by their sum (*I* ^−^ +  *I* ^+^). The analysis of the exchange interaction (*J*) is based on Weiss theory and follows a self‐consistent fitting procedure for the experimental magnetization curves, as proposed by Faraggi et al.^[^ ^45^ ^]^ Under the assumption of a homogeneous magnetization and the approximation of the effective bandwidth (W) to the Fermi level density of the states, W and J remain initially unknown. By fixing the temperature (*T*), Landé g‐factor, and spin quantum number (*S*), a pair of *J* and *W* values is determined to best reproduce the experimental magnetization trend.

### Theoretical Methods

Ground state density functional theory (DFT) calculations were conducted using the Vienna Ab‐initio Simulation Package (VASP) version 5.4.4 on VSC‐4.^[^ ^53^, ^54^ ^]^ Exchange‐correlation effects were treated using the Perdew–Burke–Enzerdorf generalized gradient approximation (PBE‐GGA),^[^ ^55^ ^]^ supplemented with a Grimme D3 vdW‐correction with Becke‐Johnson damping.^[^ ^56^ ^]^ To accurately represent hybridization, a self‐interaction error correction for strongly localized d‐orbitals on the Ni atoms was used with an effective Hubbard‐U parameter of 3 eV in the Dudarev ansatz.^[^ ^57^ ^]^ Starting with experimentally determined structures, all systems were fully relaxed until atomic forces were below 0.01 eV Å^−1^. The interface was modeled using the repeated slab approach with a 25 Å vacuum layer and a dipole layer within the vacuum region to address electric field discrepancies between the upper and lower side of the slab.^[^ ^58^ ^]^ The silver substrate bulk was modeled with five layers, allowing relaxation only in the top two layers during geometry optimization. The Γ‐centered grid sampling of the first Brillouin zone was 4 × 4 × 1 and 6 × 6 × 1 for the LC and HC phase, respectively. For the geometry relaxation a coarser grid of 2 × 2 × 1 and 4 × 4 × 1 was used.

## Conflict of Interest

The authors declare no conflict of interest.

## Supporting information



Supporting Information

## Data Availability

The data that support the findings of this study are available from the corresponding author upon reasonable request.
