# Supplementary material for: Substrate Stabilized Charge Transfer Scheme In Coverage Controlled 2D Metal Organic Frameworks
Source: Small. 2025 Feb 17;21(12):2500507. doi: 10.1002/smll.202500507 (PMC11947512; doi:10.1002/smll.202500507)
Supplement: Supplementary file 1 — Supporting Information [file SMLL-21-2500507-s001.pdf]

NANO · MICRO
small

Supporting Information

for *Small*, DOI 10.1002/smll.202500507

Substrate Stabilized Charge Transfer Scheme In Coverage Controlled 2D Metal Organic Frameworks

Simone Mearini, Dominik Brandstetter*, Yan Yan Grisan Qiu, Daniel Baranowski, Iulia Cojocariu, Matteo Jugovac, Pierluigi Gargiani, Manuel Valvidares, Luca Schio, Luca Floreano, Andreas Windischbacher, Peter Puschnig*, Vitaliy Feyer* and Claus Michael Schneider*

Supporting Information

Substrate Stabilized Charge Transfer Scheme in Coverage Controlled 2D Metal Organic Frameworks

Simone Mearini, Dominik Brandstetter,* Yan Yan Grisan Qiu, Daniel Baranowski, Iulia Cojocariu, Matteo Jugovac, Pierluigi Gargiani, Manuel Valvidares, Luca Schio, Luca Floreano, Andreas Windischbacher, Peter Puschnig,* Vitaliy Feyer* and Claus Michael Schneider*

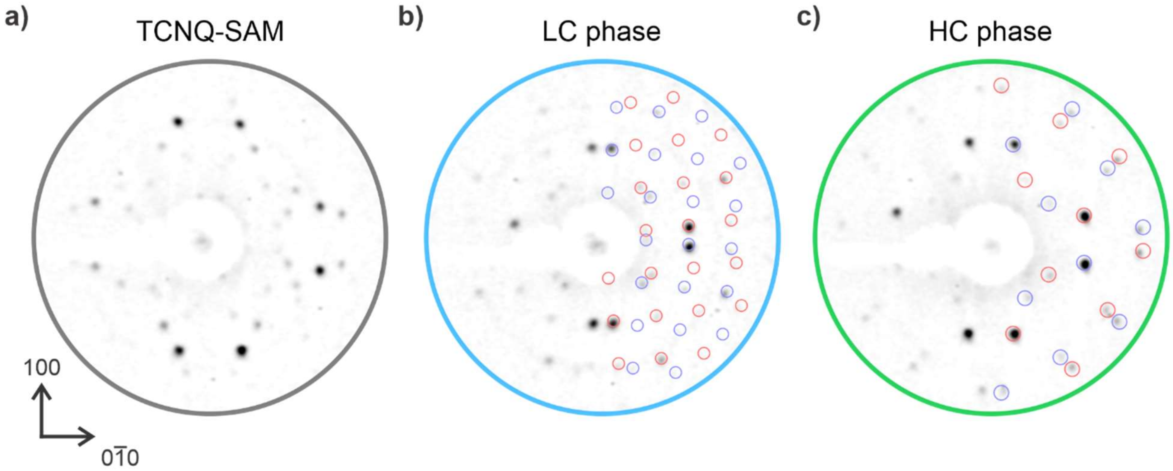

Figure S1. Experimental LEED patterns acquired with an incident electron beam energy of 15 eV for the a) TCNQ-SAM, b) LC and c) HC phase; the colored circles in b) and c) represent the simulated LEED patterns obtained by starting from the matrices (5, 4; -4, 5) and (4, -1; -1, -4) for the b) LC and c) HC phase, respectively; the distinct colors (red and blue) indicate the two domains, mirrored with respect to the [100] direction.

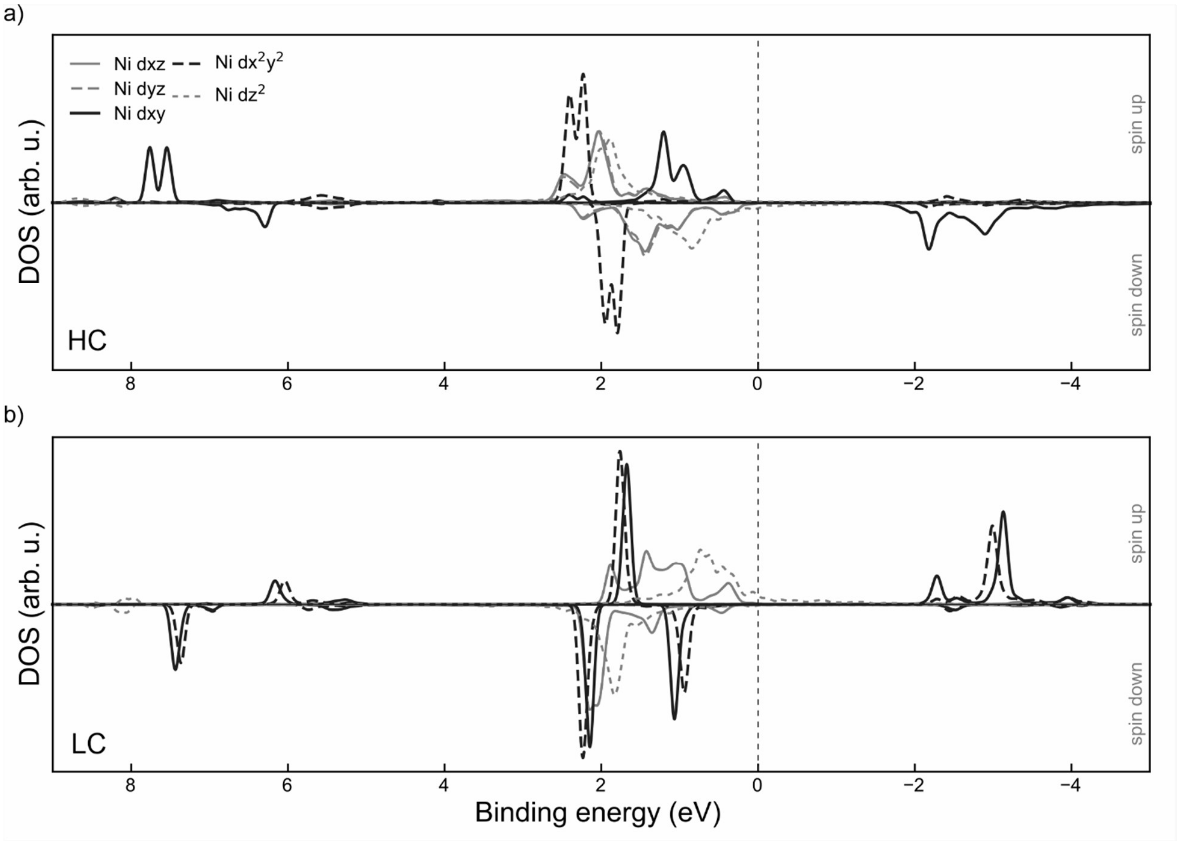

Figure S2. Calculated DOS projected on all individual Ni 3d states for a wide *BE* range for the a) HC and b) LC phase, respectively.

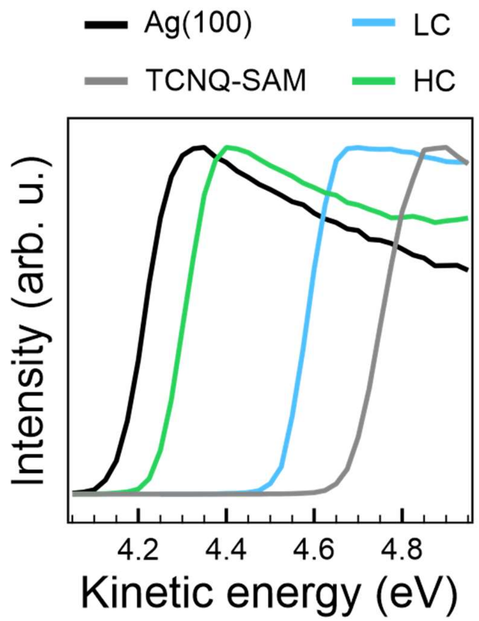

Exp.	Φ (eV)	$Φ_{rel}$ (eV)
Ag(100)	4.15	0.00
TCNQ-SAM	4.68	+0.53
LC	4.52	+0.37
HC	4.26	+0.11

Sim.	Φ (eV)	$Φ_{rel}$ (eV)
Ag(100)	4.20	0.00
TCNQ-SAM	4.86	+0.66
LC	4.72	+0.52
HC	4.56	+0.36

Figure S3. Experimental Φ spectra (photon energy 30 eV, p-polarized light) acquired for the Ag(100), TCNQ-SAM, LC and HC phase, represented by the black, grey, cyan and green lines, respectively; the tables report the Φ and Φ_{rel} values, determined experimentally (top) and theoretically (bottom) for the studied systems. The Φ_{rel} values are evaluated as the difference between the system Φ and the Ag(100) Φ.

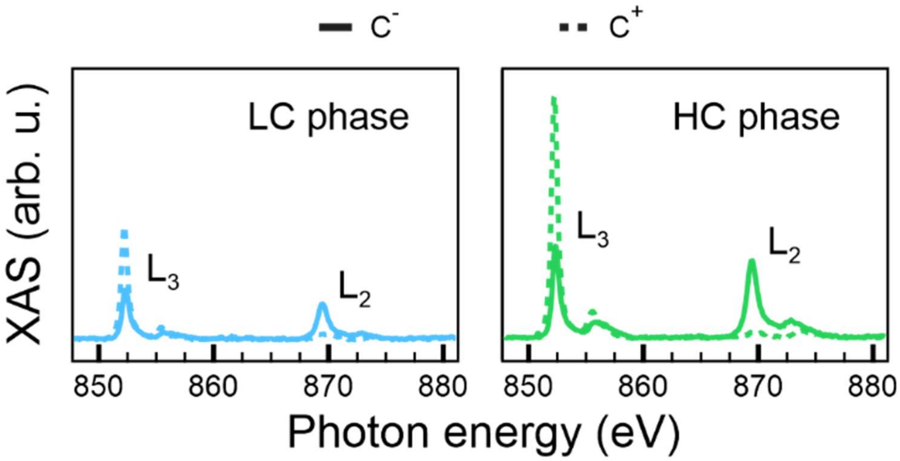

Figure S4. XAS spectra acquired across the Ni $L_{2,3}$-edges with circular left (solid line) and circular right (dashed line) polarized light of the HC (green curves) and LC (cyan curves) Ni MOFs obtained at normal incidence geometry ($T \sim 5$ K, $B = 6$ T); "L_3" and "L_2" indicate the main absorption peaks.

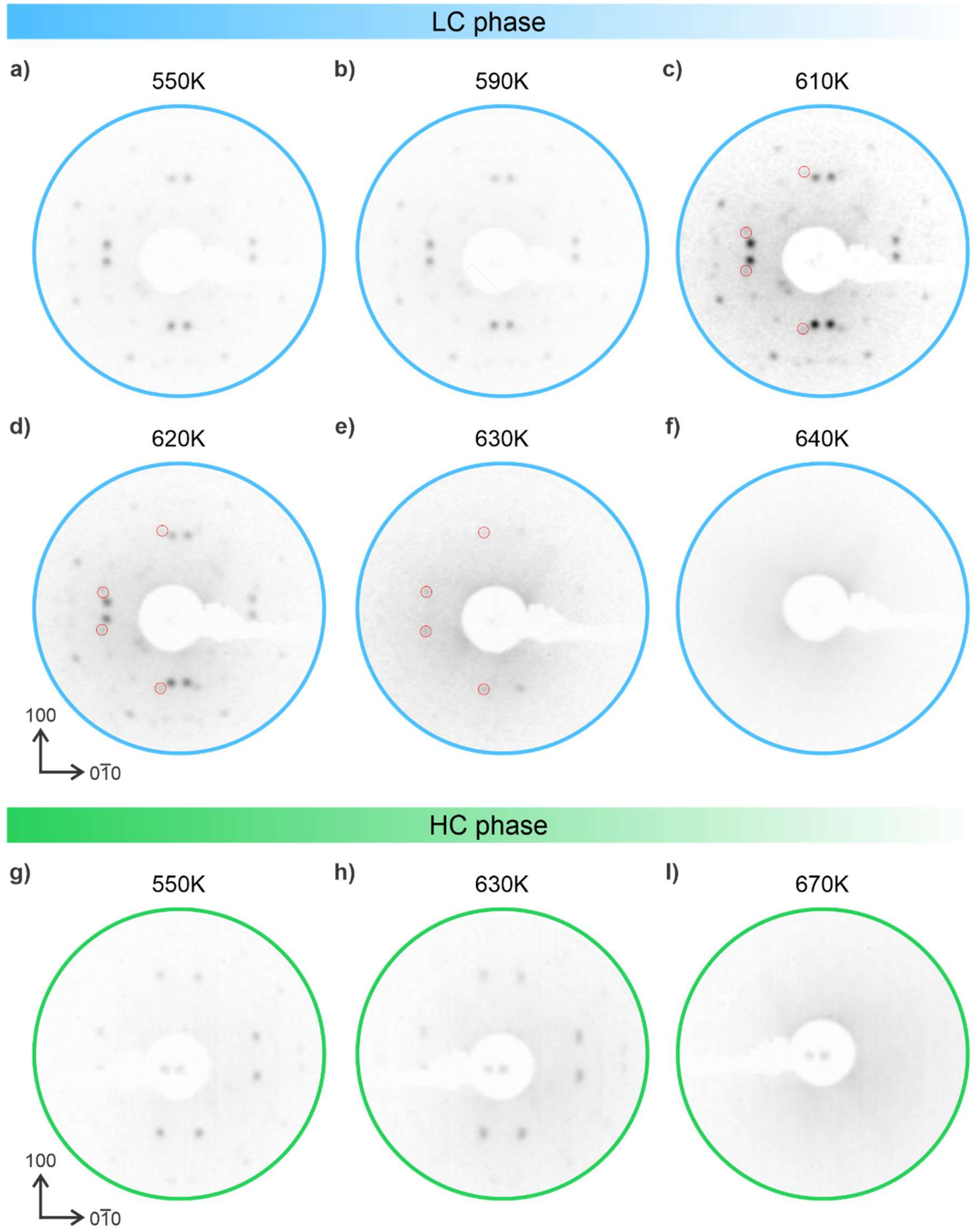

Figure S5. Experimental LEED patterns acquired with an incident electron beam energy of 15 eV at increasing temperature for the a-f) LC phase and g-l) HC phase. The red dots indicate the new phase appearing in the LC phase at 610 K.
